# Aberrant ATRX protein expression is associated with poor overall survival in NF1-MPNST

**DOI:** 10.18632/oncotarget.25195

**Published:** 2018-05-01

**Authors:** Hsiang-Chih Lu, Vanessa Eulo, Anthony J. Apicelli, Melike Pekmezci, Yu Tao, Jingqin Luo, Angela C. Hirbe, Sonika Dahiya

**Affiliations:** ^1^ Division of Neuropathology, Department of Pathology and Immunology, Washington University School of Medicine, St. Louis, MO, USA; ^2^ Department of Medicine, Washington University School of Medicine, St. Louis, MO, USA; ^3^ Department of Radiation Oncology, Washington University School of Medicine, St. Louis, MO, USA; ^4^ Siteman Cancer Center, Washington University School of Medicine, Saint Louis, MO, USA; ^5^ Department of Pathology, University of California San Francisco School of Medicine, San Francisco, CA, USA; ^6^ Siteman Cancer Center Biostatistics Shared Resource, Division of Public Health Sciences, Department of Surgery, Washington University School of Medicine, St. Louis, MO, USA; ^7^ Division of Medical Oncology, Washington University School of Medicine, St. Louis, MO, USA

**Keywords:** neurofibromatosis, MPNST, plexiform neurofibroma, atypical neurofibroma, ATRX

## Abstract

Malignant Peripheral Nerve Sheath Tumors (MPNSTs) are aggressive soft tissue sarcomas that can occur sporadically or in the setting of the Neurofibromatosis type 1 (NF1) cancer predisposition syndrome. These tumors carry a dismal overall survival. Previous work in our lab had identified ATRX chromatin remodeler (*ATRX*), previously termed, Alpha Thalassemia/Mental Retardation Syndrome X Linked as a gene mutated in a subset of MPNSTs. Given the great need for novel biomarkers and therapeutic targets for MPNSTs, we sought to determine the expression of ATRX in a larger subset of sporadic and NF1 associated MPNSTs (NF1-MPNSTs). We performed immunohistochemistry (IHC) on 74 MPNSTs (43 NF1-associated and 31 sporadic), 21 plexiform neurofibromas, and 9 atypical neurofibromas. Using this approach, we have demonstrated that 58% (43/74) of MPNSTs have aberrant ATRX expression (<80% nuclear expression) compared to only 7% (2/30) of benign (plexiform and atypical) neurofibromas. Second, we demonstrated that 65% (28/43) of NF1-MPNSTs displayed aberrant ATRX expression as did 48% (15/31) of sporadic MPNSTs. Finally, we show that aberrant ATRX expression was associated with a significantly decreased overall survival for patients with NF1-MPNST (median OS of 17.9 months for aberrant expression and median OS not met (>120 months) for intact expression, *p* = 0.0276). In summary, we demonstrate that ATRX is aberrantly expressed in the majority of NF1-MPNSTs, but not plexiform or atypical neurofibromas. Additionally, aberrant ATRX expression is associated with decreased overall survival in NF1-MPNST, but not sporadic MPNST and may serve as a prognostic marker for patients with NF1-MPNST.

## INTRODUCTION

Sarcomas are rare tumors that usually arise from bone or soft tissue. Among soft tissue sarcomas, over 70 different subtypes exist. MPNSTs are aggressive sarcomas that account for approximately 5% of all soft tissue sarcomas [1]. Almost half of malignant peripheral nerve sheath tumors (MPNSTs) occur sporadically (in the absence of a tumor predisposition syndrome) or as a complication of prior radiation therapy and the other half occur in the setting of the Neurofibromatosis type 1 (NF1) tumor predisposition syndrome [2]. These tumors are thought to be composed of malignant cells with Schwannian differentiation, and in the setting of NF1 often arise from a benign precursor lesion, a plexiform neurofibroma (benign neurofibroma involving multiple fascicles of a peripheral nerve) or atypical neurofibromas (benign neurofibroma with cytologic atypia, increased cellularity, scattered mitotic figures, and/or loss of neurofibroma architecture, albeit with a low mitotic index of < 3/10 HPFs [3]). Even with multi-modality therapy, the overall prognosis for MPNSTs is dismal with 50% of patients failing to survive beyond five years of diagnosis [4–6]. Prior genomic studies in our laboratory aimed at identifying potential therapeutic targets and biomarkers for MPNSTs had identified *ATRX* as a gene mutated in a subset of MPNSTs [7].

ATRX is a member of the SWI/SNF family of DNA helicases that plays a role in chromatin regulation and maintenance of telomeres. It regulates incorporation of histone H3.3 into telomeric chromatin [8, 9]. The protein product is also implicated in the initiation of non-homologous end joining (NHEJ) in gliomas [10] , a process by which double strand DNA breaks (DSB) are repaired. When disrupted, NHEJ deficiency results in genome instability, cell cycle arrest, and/or cellular death; however in tumor cell lines that lack cell cycle checkpoints (e.g. p53 mutant), it can actually lead to a mutator phenotype and promote tumor progression [11]. The loss of ATRX function also contributes to alternative lengthening of telomeres (ALT), a telomerase independent mechanism of telomere lengthening leading to cellular immortality, thus also promoting tumorigenesis [12]. Further, ATRX is also known to direct members of the polycomb repressor complex 2 (PRC2) to target promoters and aid in heterochromatin formation and epigenetic silencing, another way in which it may regulate tumorigenesis [13].

Given the recent data suggesting a role for ATRX in the pathogenesis and prognostication of GBM [14] and other gliomas, [11, 15] including NF1-related gliomas [16], and our recent data demonstrating *ATRX* mutations in 2/7 MPNSTs sequenced cases [7], we sought to determine whether or not ATRX expression was altered in a larger subset of MPNSTs. Most reported mutations in *ATRX* are inactivating mutations and thus lead to loss of protein expression. As such, loss of ATRX expression is often thought of as a surrogate for mutational analysis. Studies in other malignancies have shown a significant correlation between *ATRX* mutational status and a mosaic pattern of staining. For example 66% of pancreatic cancer cases and 40% of gliomas with a mosaic staining pattern had an identifiable *ATRX* mutation identified by next-generation sequencing [15, 17, 18]. It is possible that another mechanism such as methylation changes lead to silencing of ATRX expression in the other mosaic cases. In the current study, we analyzed ATRX expression by immunohistochemistry in 104 tumor samples (74 MPNST samples from both sporadic and NF1 patients as well as 21 plexiform neurofibromas and 9 atypical neurofibromas) from a total of 97 different patients. Of note, there are 104 tumors from 97 patients as seven patients had tissue available from both their plexiform neurofibroma and the subsequent MPNST that arose within the same anatomic location.

## RESULTS

We collected data and specimens from 104 tumors including 74 MPNSTs, 9 atypical neurofibromas, and 21 plexiform neurofibromas coming from a total of 97 patients. Seven patients were represented in both the plexiform neurofibroma group and the MPNST group. Of the patients from the Washington University cohort 11/21 NF1-associated MPNSTs clearly arose from a precursor plexiform or diffuse NF based on assessment of the pathologic specimen. The other 10 NF1 patients did not have imaging prior to their diagnosis of MPNST and the tumor specimen consisted entirely of high grade MPNST with no evidence of a pre-existing benign component; these cases were assumed to have arisen *de novo*. None of the sporadic MPNSTs showed any evidence of a benign precursor in the pathologic assessment (Table 1). This information is not available for the UCSF cohort. Additionally, one sporadic MPNST arose in an area of prior radiation treatment and none of the NF1-associated MPNSTs in either cohort arose in the area of prior radiation field (Table 1). Most patients in this study were over the age of 18 years at diagnosis. Of the patients with PNs (*n* = 21), 33% (7/21) were prepubertal. As expected, only one (1/11) pediatric patient developed an MPNST, and the rest of the pediatric patients in the study had plexiform neurofibromas, as it is very uncommon for an MPNST to present in childhood. The median age at diagnosis was 34 years of age (23 years of age for plexiform and atypical neurofibromas and 39 years of age for MPNSTs). Approximately 2/3^rds^ of the patients had NF1 (43/74 MPNSTs, 7/9 atypical neurofibromas, and 21/21 plexiform neurofibromas) (Tables 2 and 3).

**Table 1:** The origin of MPNSTs

	NF1-associated	Sporadic
Known plexiform NF precursor	11/21	0/19
Radiation induced	0/21	1/19
*De novo*	10/21	18/19

Data available only for the Washington University cohort (21 NF1-associated and 19 sporadic MPNSTs).

**Table 2:** Clinical characteristics of MPNST patients

Characteristic	ATRX positive (*N* = 31)	ATRX mosaic (*N* = 29)	ATRX negative (*N* = 14)	Total (*N* = 74)
**Sex—no. (%)**				
Male	13 (42)	12 (41)	8(57)	33 (45)
Female	18 (58)	17 (59)	6 (43)	41 (55)
**Age at diagnosis—yr**				
Median	38	39	36	39
Range	18-79	22-71	11-58	11–79
**Age Category—no. (%)**				
<18 yo	0	0	2 (14)	2 (3)
18 to 65 yo	27 (87)	28 (97)	12 (86)	67 (90)
>65 yo	4 (13)	1 (3)	0	5 (7)
**NF Status—no. (%)**				
NF1	15 (48)	18 (62)	10 (71)	43 (58)
Sporadic	16 (52)	11(38)	4 (29)	31(42)

no = number, yr = year, NF = Neurofibromatosis.

**Table 3:** Clinical characteristics of atypical and plexiform neurofibromas

Characteristic	ATRX positive (*N* = 28)	ATRX mosaic (*N* = 2)	Total (*N* = 30)
**Sex—no. (%)**			
Male	10 (36)	1 (50)	11 (37)
Female	18 (64)	1 (50)	19 (63)
**Age at diagnosis—yr**			
Median	21	11	23
Range	7–67	4–27	4–67
**Age Category—no. (%)**			
<18 yo	9 (32)	1 (50)	10 (33)
18 to 65 yo	18 (64)	1 (50)	19 (63)
>65 yo	1 (4)	0	1 (4)
**NF Status—no. (%)**			
NF1	26 (93)	2 (100)	28 (94)
Sporadic	1 (3)	0	1 (3)
Not Reported	1 (3)	0	1 (3)
**Tumor Type—no.(%)**			
Atypical Neurofibroma	9 (32)	0	9 (30)
Plexiform Neurofibroma	19 (68)	2 (100)	21 (70)

There were no ATRX negative patients in the above tumor types.

**Figure 1:**
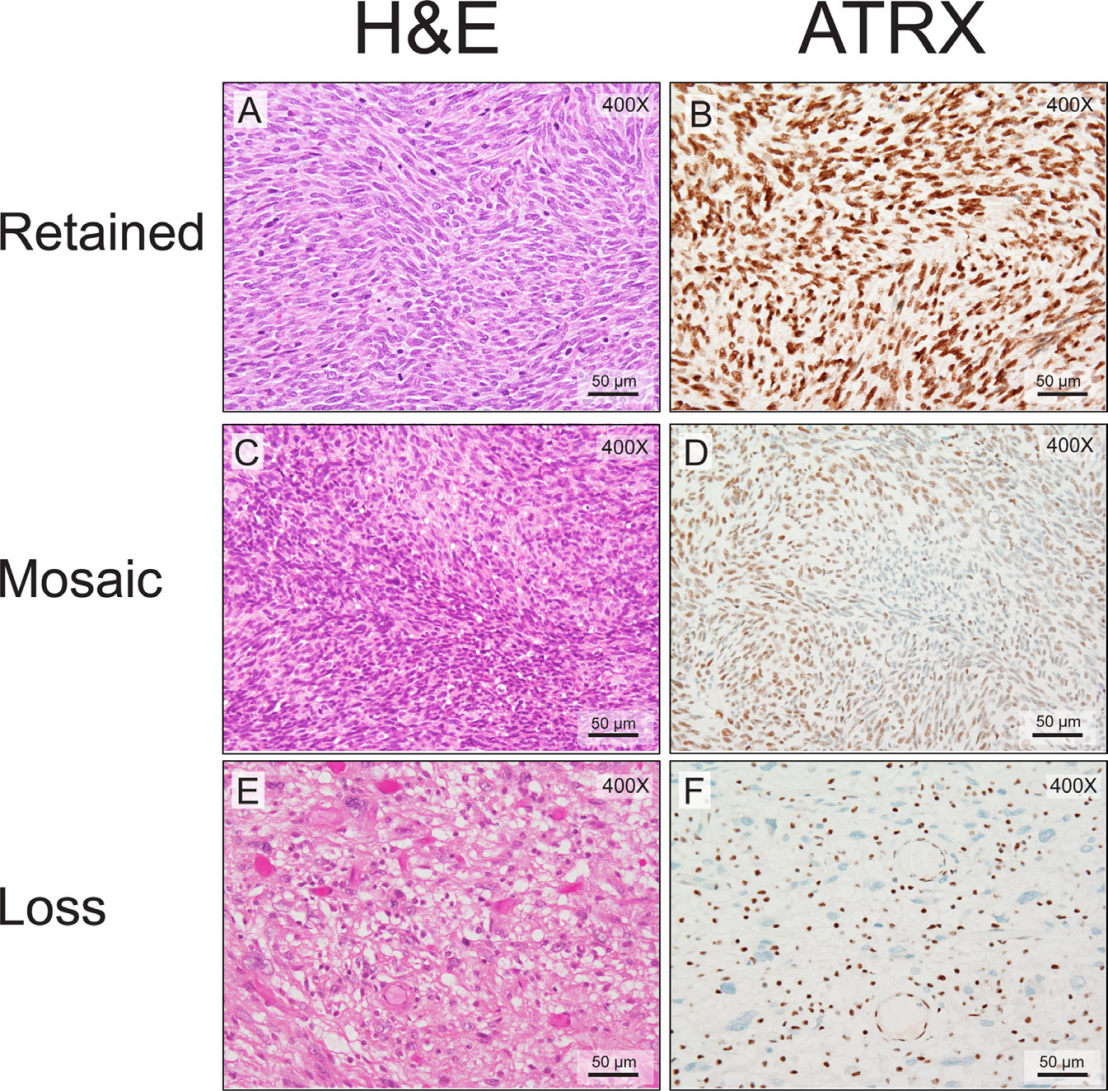
MPNSTs show three distinct patterns of ATRX staining Routine hematoxylin and eosin staining demonstrates typical MPNST morphology. (**A**, **C** and **E**). ATRX nuclear staining in the tumor cells can be classified as retained (**B**, 81–100%), mosaic (**D**, 21–80%), or negative (**F**, 0–20%). Note the residual ATRX nuclear staining in the inflammatory and endothelial cells in panel F as an internal positive control. The mitotic indices are 62/10 HPFs (A and B), 6/10 HPFs (C and D), and 4/10 HPFs (E and F).

We next wanted to determine the ATRX expression pattern in MPNSTs. MPNSTs fell into three groups by immunohistochemical staining: retained, mosaic, or complete loss (Figure 1). This is similar to what has been previously published in adult diffuse gliomas [15]. Of note, loss of ATRX staining has been associated with *ATRX* mutational status [19] and mosaic staining has been associated with *ATRX* mutations in approximately 40% of cases [15]. As can be seen in Table 1, slightly more than half of the MPNSTs (43/74 tumors) examined exhibited either a mosaic pattern (29 tumors) or loss of ATRX (14 tumors). For 6 of the cases we did have sequencing data as well. Of those cases 2/6 cases harbored a non-synonymous mutation in *ATRX* and both of these cases exhibited a loss of ATRX by IHC. There was no association with other common genes mutated in MPNSTs in this small subset (Supplementary Table 1).

**Figure 2:**
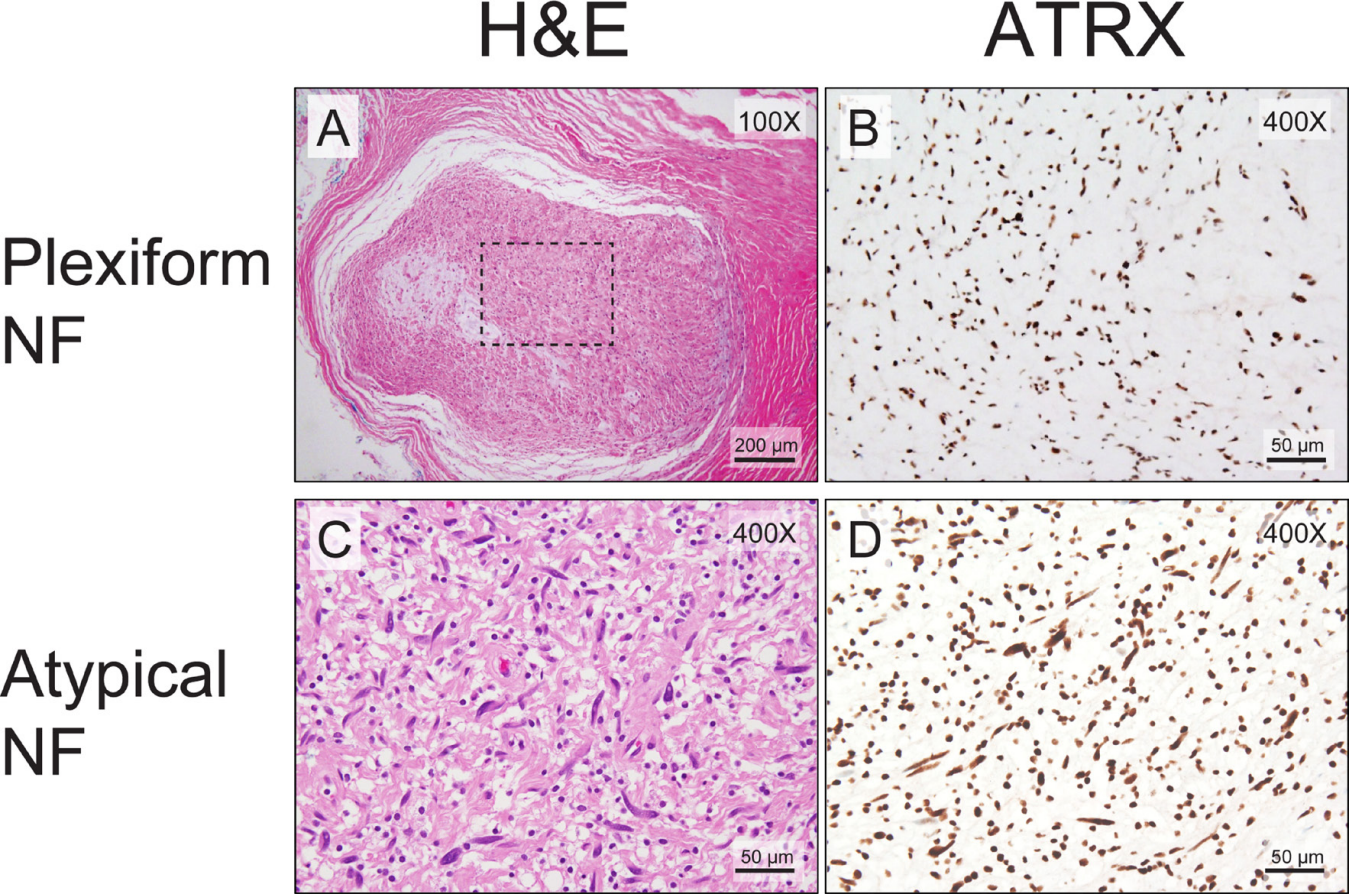
Almost all plexiform neurofibromas show retained ATRX staining pattern Low-power view of the routine hematoxylin and eosin staining demonstrates typical plexiform neurofibroma morphology (**A**). The ATRX immunostain shows retained nuclear staining (**B**). All atypical neurofibromas show retained ATRX staining pattern. A high-power view of the routine hematoxylin and eosin staining demonstrates an atypical neurofibroma (**C**). The ATRX immunostain shows retained nuclear staining (**D**). The mitotic index for the atypical neurofibroma in C and D is <1/50 HPFs.

**Figure 3:**
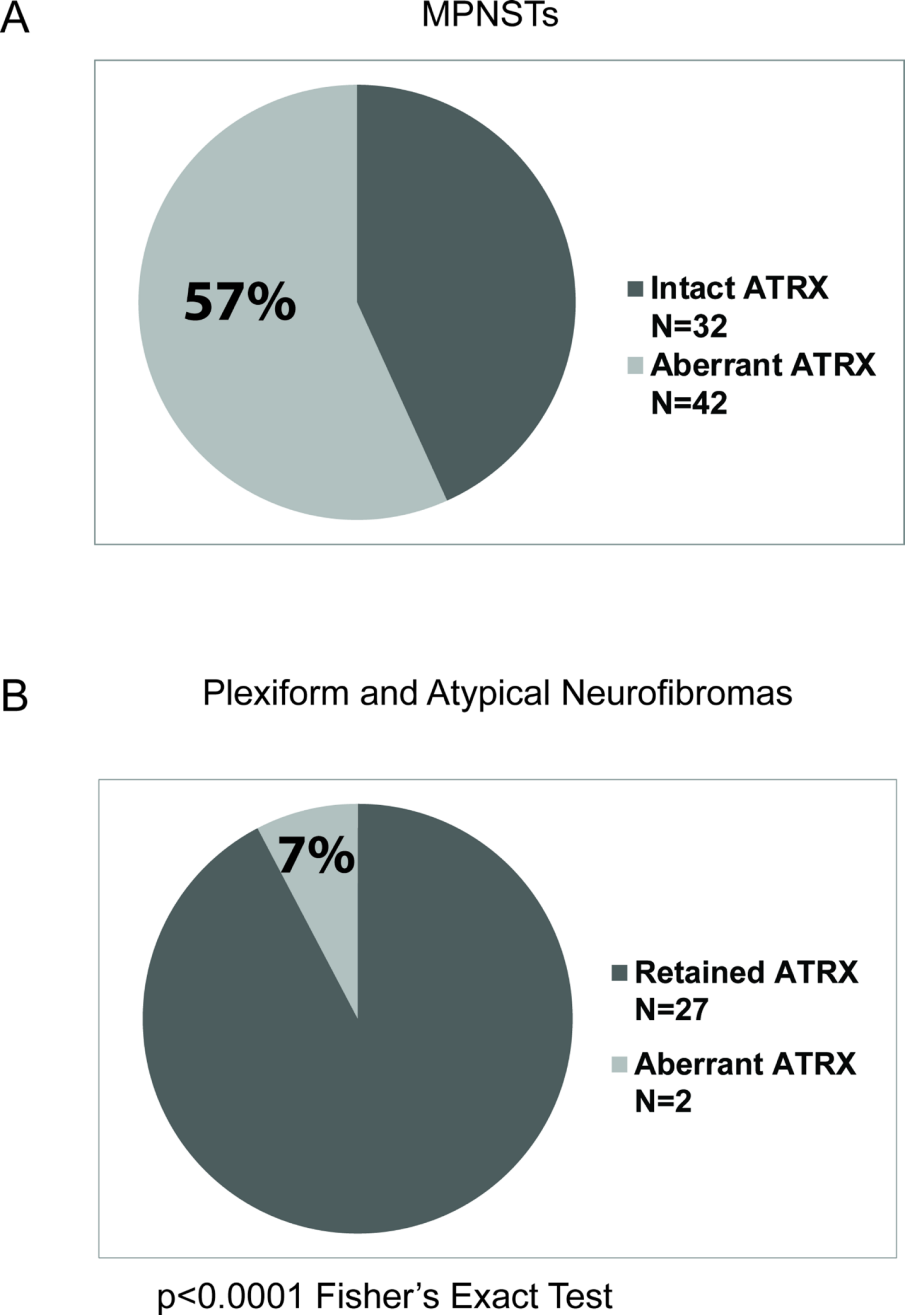
Higher percentage of MPNSTs show aberrant ATRX staining pattern compared to plexiform neurofibromas The number of cases with either retained or aberrant ATRX staining patterns are plotted. MPNSTs are depicted in panel (**A**) and plexiform neurofibromas are depicted in panel (**B**). The percentage of MPNSTs with aberrant ATRX staining pattern is significantly different from that of plexiform neurofibromas. (*p* = 0.0011, Fischer's Exact Test).

We then examined whether aberrant ATRX protein expression was also seen in plexiform and atypical neurofibromas, known precursor lesions to MPNSTs. Interestingly, only 7% (2/30) of plexiform neurofibromas and atypical neurofibromas show aberrant ATRX protein expression (Figure 2), compared to 57% (43/74) of MPNSTs showing aberrant ATRX protein (Figure 3, *p* < 0.0001, Fisher’s Exact Test). Taken together, these data suggest that changes in levels of ATRX are associated with malignancy, rather than the pre-malignant state.

**Figure 4:**
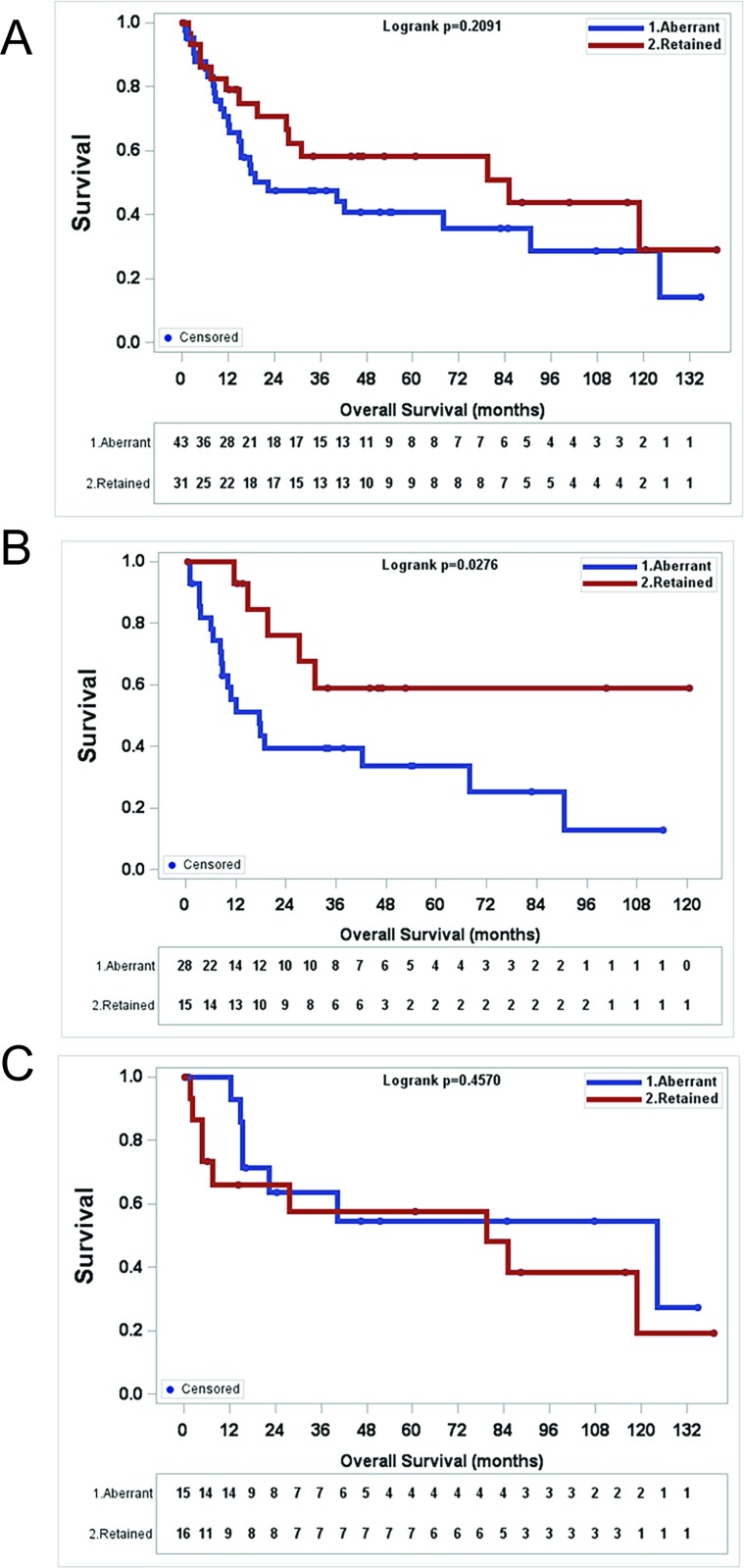
Aberrant ATRX staining pattern (mosaic or negative) in MPNST correlates with worse survival in NF1 patients (**A**) There is no significant survival difference with aberrant MPNST staining pattern in all MPNST patients. (*p* = 0.2091), Kaplan-Meier analysis and log-rank test). (**B**) In NF1 patients, aberrant ATRX staining pattern in MPNSTs correlates with worse overall survival. (*p* = 0.0276), Kaplan-Meier analysis and log-rank test) (**C**) In patients with sporadic MPNSTs, ATRX staining pattern does not correlate with survival. (*p* = 0.4570), Kaplan-Meier analysis and log-rank test).

Finally, we wanted to determine whether or not ATRX expression correlated with overall survival (OS). MPNSTs with aberrant ATRX expression showed a trend toward worse OS in comparison to MPNSTs with retained ATRX expression, though statistically not significant (Figure 4A, *p* = 0.2091, log rank test). However, when we broke the cohort into NF1-MPNSTs and sporadic MPNSTs, we did observe a statistically significant decrease in OS for patients with NF1-MPNSTs who have aberrant ATRX expression (Figure 4B, *p* = 0.0276, log rank test), but there is no difference in overall survival based on ATRX status for sporadic MPNSTs (Figure 4C, *p* = 0.4570, log rank test). In a multivariate analysis of NF1-MPNSTs which evaluated margin status, adjuvant and neoadjuvant treatment, age, initial tumor size, presence of metastasis at diagnosis, initial location of the tumor, and gender, aberrant ATRX expression was found to be an independent poor prognostic factor with a hazard ratio of 5.3 (95%CI:1.367–20.433) (Table 4).

**Table 4:** Multivariate overall survival analysis of NF1-MPNSTs

Parameter	*P*-value	Hazard ratio	95% Hazard ratio confidence limits	
**ATRX(Aberrant vs. Retained)**	0.0158	5.284	1.367	20.433
**Margin Status (Positive vs. Negative)**	0.0583	2.742	0.965	7.792
**Metastasis at Diagnosis (Yes vs. No)**	0.0009	11.863	2.758	51.026
**Adjuvant or Neoadjuvant Treatment (Yes vs No)**	0.6196	0.659	0.127	3.425
**Age**	0.6654	0.989	0.940	1.040
**Tumor Size (<5 cm or >5 cm)**	0.2236	1.088	0.950	1.246
**Tumor location (extremity vs. non-extremity)**	0.9895	0.990	0.228	4.306
**Gender**	0.9984	1.001	0.299	3.352

## DISCUSSION

MPNSTs are aggressive sarcomas with limited treatment options and poor overall survival. Furthermore, there are few clinicopathological features that influence outcome and no accurate biologic markers to predict the clinical course or progression. As such, there is a pressing need to identify new therapeutic targets and biomarkers of disease biology. Given the recent literature suggesting a role for ATRX in the pathogenesis and prognostication for GBM [14] and other gliomas, [11, 15] including NF1-related gliomas [16], our recent publication demonstrating *ATRX* mutations in 2/7 MPNSTs sequenced [7], and the reported correlation between ATRX immunostaining and mutational status [15, 17, 18], we explored whether or not ATRX expression was altered in a large subset of MPNSTs.

Using IHC, we have demonstrated that 57% (43/74) of MPNSTs have aberrant ATRX expression compared to only 10% (2/21) of plexiform neurofibromas, and no (0/9) atypical neurofibromas, benign precursor lesions to MPNSTs. These data suggest that changes in ATRX expression are associated with malignancy, but not pre-malignant disease. This is in stark contrast to two previously published studies of ATRX loss in a variety of sarcoma subtypes [20, 21], which demonstrated loss of ATRX in 1/17 (6%) and 2/47 (4%) of MPNST samples, respectively. However, it should be noted that these studies gave no breakdown of sporadic vs. NF1 associated MPNST. In our study, 28 of 43 (65%) MPNSTs with aberrant ATRX expression (mosaic or absent expression) were NF1 associated (Table 2). Given that NF1-associated MPNSTs represented 65% of our cohort, this may explain the discrepancy. Furthermore, in the very recently published TCGA analysis of the genetic and epigenetic landscape of soft tissue sarcomas, 2 of the 5 MPNST samples were noted to have copy number loss of the *ATRX* locus (40%), a number more in line with the 60% aberrant expression noted in the current study [22].

Most intriguingly, we found that there was a significant decrease in overall survival for patients with NF1-MPNSTs who have aberrant ATRX expression (*p* = 0.04, log rank test) (Figure 4B), while there is no difference in overall survival based on ATRX status for sporadic MPNSTs (Figure 4C). Furthermore, ATRX status also was found to independently predict overall survival in our multivariate analysis of NF1-MPNSTs. We know that the biology of NF1-MPNSTs is different from that of sporadic MPNSTs as the survival is worse for NF1-MPNSTs compared to sporadic MPNSTs [5, 23]. Additionally, different molecular features are seen in NF1 vs sporadic MPNSTs. For example, 88% of NF1-MPNSTs exhibit loss of neurofibromin expression, but this is only true for 43% of sporadic MPNSTs [24]. Similarly loss of tri-methylation at lysine 27 of histone 3 (H3K27me3) is seen in 95% of sporadic MPNSTs compared to only 60% of NF1-associated MPNSTs [25]. Taken together these data support the notion that the biology of NF1-MPNSTs and sporadic MPNSTs is different and suggest that aberrant ATRX expression may be cooperating with some other alteration in NF1-MPNSTs leading to a poorer prognosis for these tumors.

While *ATRX* is an X-linked tumor suppressor, our data support the notion that it is a cooperating mutation in both NF1-associated MPNSTs as well as sporadic MPNSTs, rather than an initiating mutation in tumorigenesis, as our data show a much higher percentage of MPNSTs have aberrant expression by IHC versus the precursor plexiform neurofibromas. In addition to inactivation and loss of heterozygosity (LOH) via the usual mechanisms (mutations, deletions, epigenetic silencing, etc), X-linked tumor suppressors are also vulnerable to inactivation via skewed X chromosomal inactivation and bi-allelic epigenetic silencing in females, as either the paternal or maternal X chromosome is wholly inactivated during embryogenesis in each progenitor cell, and this is passed on to its progeny, including differentiated daughter cells [26]. Thus, on the surface, while it might seem as though a sex bias would exist for *ATRX*, this is not seen due to the fact that both males and females truly only have one functioning copy of *ATRX* per cell, and thus both sexes have an equal likelihood of developing LOH or inactivating mutations in the one functional copy. This is borne out by the data presented in Table 2, which shows no statistically significant difference between sex and ATRX staining pattern by Fisher’s exact test (*p* = 0.61). Furthermore, it should be noted that individuals with germline inactivating mutations or deletions in *ATRX*, do not have a cancer predisposition syndrome, even in the male population who manifest the alpha thalassemia/mental retardation X-linked syndrome.

Given the marked difference in survival mentioned above, future work will focus on whether reduced ATRX expression results in a cell-autonomous growth advantage in NF1-MPNSTs versus sporadic MPNSTs, and whether such a difference requires NF1 loss or downstream pathway activation. Furthermore, ATRX is also known to direct members of the polycomb repressor complex 2 (PRC2) to target promoters and aid in heterochromatin formation and epigenetic silencing [13]. Intriguingly, recent reports have demonstrated a prominent association of inactivating mutations in EED and SUZ12, two members of the PRC2, with the development of both sporadic and NF1-associated MPNSTs [27, 28]. We plan to investigate whether or not ATRX also plays a role in the epigenetic silencing of certain PRC2-targeted genes, which may be amenable to future therapeutic strategies.

Finally, ATRX is also postulated to play a role in the alternative lengthening of telomeres (ALT), which is dependent on homologous recombination of sister chromatids in G2/M phase or stalled replication forks in S phase [29, 30]. Thus, future work will also hinge on whether ATRX is aberrantly lost in other subtypes of sarcomas as well as MPNSTs. For example, loss of ATRX and subsequent activation of ALT has been observed in liposarcomas and correlates with poor overall survival [31]. However, its role in many other subtypes of soft tissue sarcoma remains unclear. Changes in ATRX expression, therefore, may be able to serve as a biomarker for MPNSTs and even other types of sarcomas.

In summary, this study represents the first comprehensive look at ATRX expression in MPNSTs. Herein, we show that ATRX is aberrantly expressed in the majority of MPNSTs and that this aberrant expression is associated with poor overall survival in NF1-associated MPNSTs, which suggests that ATRX expression could be evaluated as a prognostic biomarker for these aggressive sarcomas.

## MATERIALS AND METHODS

### Patient selection

This study was performed under active Human Studies Protocols approved by the Institutional Review Boards at each respective institution in accordance with the 1964 Helsinki Declaration and its later amendments or comparable ethical standards. Patients with a diagnosis of malignant peripheral nerve sheath tumor (MPNST), plexiform neurofibroma, or atypical neurofibroma between 2011 and 2017 were retrieved from the neurofibromatosis type 1 (NF1) patient database or the sarcoma database from the Department of Hematology and Oncology of Washington University in St Louis. Patients with a diagnosis of NF1 had been followed in the NF1 clinic. Patients called sporadic had either been seen in the NF1 clinic and deemed not to have NF1, evaluated by an NF1 specialist in the sarcoma clinic and deemed not to have NF1, or had a well-documented skin exam, musculoskeletal exam, and family history in the medical record that would allow a physician to conclude that the patient had no clinical diagnostic features of NF1. All cases from Washington University were re-evaluated by one of the authors (SD) in addition to the clinical pathologist who made the initial diagnosis on the case. Whole tissue sections from surgical excisions were evaluated for the 40 MPNST cases, 21 plexiform neurofibroma cases, and 9 atypical neurofibroma cases from Washington University. Patients with MPNST diagnosed at UCSF were identified from Anatomic Pathology archives between 1990 and 2012 for generation of a tissue microarray (TMA). A chart review was performed at the time the TMA was generated. If a clinical diagnosis of NF1 was made and documented in the chart, the MPNST was deemed to be NF1-associated. If the chart documentation stated that there were no clinical features of NF1, the MPNST was deemed to be sporadic. The previously-generated tissue microarrays which included 34 patients treated at UCSF were utilized in this study. The cores were obtained from the most representative area of tumor and two 2 mm cores from each tumor were evaluated. All cases chosen for the TMA were evaluated by one of the authors (MP) as well as two other pathologists at UCSF [32]. Information on patients’ sex, age, NF1 status, and survival data were obtained from the electronic medical record and Social Security Death Index.

### ATRX immunohistochemistry and quantification

The hematoxylin and eosin–stained sections were retrieved, and were reviewed to confirm the diagnosis. Additional formalin-fixed paraffin-embedded sections were obtained from the patient blocks at Washington University in St. Louis. UCSF cases were evaluated on the previously generated TMA. Immunohistochemical stain for ATRX (Sigma, HPA001906, 1:300, rabbit polyclonal) was performed with appropriate positive and negative controls. The slides were reviewed blinded to the patients’ NF1 status. The percentage of tumor cells showing nuclear ATRX labeling was evaluated. The ATRX IHC was evaluated semi-quantitatively across the whole tumor section used for staining. The staining results were scored semi-quantitatively, defined as lost (0–20%), mosaic (21–80%), and retained (81–100%). The mosaic and lost patterns are grouped as aberrant ATRX expression in the analysis. Mitotic index was evaluated from the most mitotically active area of the tumor (Supplementary Table 2).

### Statistical analyses

ATRX protein expression was compared between histology by Fisher’s Exact Test using Graphpad Prism Version 7.03. Patient characteristics were compared between patients with ATRX retained tumor specimens and patients with ATRX aberrant tumor sections using χ2 test or Fisher exact test as appropriate for categorical variables while non-parametric Wilcoxon rank sum test was used for continuous variables. Overall survival (OS) was defined as from date of diagnosis to date of death by any cause or date of last follow up. Date of death was obtained from medical record or Social Security Death Index, expiration note in chart or obituary. Kaplan-Meier survival curves were generated for overall survival by ATRX expression groups in NF1 MPNST and sporadic MPNST patients. The survival difference between groups was compared using the log-rank test. Raw hazard ratios (HR) and 95% CI were estimated from univariate Cox model. A multivariate Cox proportional hazards model was applied to compute adjusted HR and 95% CI after adjusting for possible prognostic variables, including margin status, adjuvant and neoadjuvant treatment, age, initial tumor size, presence of metastasis at diagnosis, initial location of the tumor, and gender. All tests were two-sided and statistical significance was defined with a *p*-value ≤ 0.05. Statistical analyses were performed with SAS (version 9.4; SAS Institute, Cary, NC) unless otherwise noted.

## SUPPLEMENTARY MATERIALS TABLES





## Abbreviations

MPNSTs: Malignant Peripheral Nerve Sheath Tumors
ATRX: ATRX chromatin remodeler
NF1-MPNSTs: NF1 associated MPNSTs
NF1: Neurofibromatosis 1
NHEJ: Non-homologous end joining
DSB: Double stranded DNA breaks
ALT: Alternative Lengthening of telomeres
GBM: Glioblastoma
PRC2: Polycomb Repressor Complex 2
H3K27me3: Histone 3 trimethylation at lysine 27
TMA: Tissue microarray
CIs: Confidence Intervals

## References

[1] Hirbe AC, Gutmann DH (2014). Neurofibromatosis type 1: a multidisciplinary approach to care. Lancet Neurol.

[2] Ducatman BS, Scheithauer BW, Piepgras DG, Reiman HM, Ilstrup DM (1986). Malignant peripheral nerve sheath tumors. A clinicopathologic study of 120 cases. Cancer.

[3] Miettinen MM, Antonescu CR, Fletcher CDM, Kim A, Lazar AJ, Quezado MM, Reilly KM, Stemmer-Rachamimov A, Stewart DR, Viskochil D, Widemann B, Perry A (2017). Histopathologic evaluation of atypical neurofibromatous tumors and their transformation into malignant peripheral nerve sheath tumor in patients with neurofibromatosis 1-a consensus overview. Hum Pathol.

[4] Hruban RH, Shiu MH, Senie RT, Woodruff JM (1990). Malignant peripheral nerve sheath tumors of the buttock and lower extremity. A study of 43 cases. Cancer.

[5] Wong WW, Hirose T, Scheithauer BW, Schild SE, Gunderson LL (1998). Malignant peripheral nerve sheath tumor: analysis of treatment outcome. Int J Radiat Oncol Biol Phys.

[6] Kourea HP, Bilsky MH, Leung DH, Lewis JJ, Woodruff JM (1998). Subdiaphragmatic and intrathoracic paraspinal malignant peripheral nerve sheath tumors: a clinicopathologic study of 25 patients and 26 tumors. Cancer.

[7] Hirbe AC, Kaushal M, Sharma MK, Dahiya S, Pekmezci M, Perry A, Gutmann DH (2016). Clinical genomic profiling identifies TYK2 mutation and overexpression in patients with neurofibromatosis type 1-associated malignant peripheral nerve sheath tumors. Cancer.

[8] Eustermann S, Yang JC, Law MJ, Amos R, Chapman LM, Jelinska C, Garrick D, Clynes D, Gibbons RJ, Rhodes D, Higgs DR, Neuhaus D (2011). Combinatorial readout of histone H3 modifications specifies localization of ATRX to heterochromatin. Nat Struct Mol Biol.

[9] Lewis PW, Elsaesser SJ, Noh KM, Stadler SC, Allis CD (2010). Daxx is an H3.3-specific histone chaperone and cooperates with ATRX in replication-independent chromatin assembly at telomeres. Proc Natl Acad Sci U S A.

[10] Koschmann C, Lowenstein PR, Castro MG (2016). ATRX mutations and glioblastoma: Impaired DNA damage repair, alternative lengthening of telomeres, and genetic instability. Mol Cell Oncol.

[11] Koschmann C, Calinescu AA, Nunez FJ, Mackay A, Fazal-Salom J, Thomas D, Mendez F, Kamran N, Dzaman M, Mulpuri L, Krasinkiewicz J, Doherty R, Lemons R (2016). ATRX loss promotes tumor growth and impairs nonhomologous end joining DNA repair in glioma. Sci Transl Med.

[12] Jafri MA, Ansari SA, Alqahtani MH, Shay JW (2016). Roles of telomeres and telomerase in cancer, and advances in telomerase-targeted therapies. Genome Med.

[13] Sarma K, Cifuentes-Rojas C, Ergun A, Del Rosario A, Jeon Y, White F, Sadreyev R, Lee JT (2014). ATRX directs binding of PRC2 to Xist RNA and Polycomb targets. Cell.

[14] Chaurasia A, Park SH, Seo JW, Park CK (2016). Immunohistochemical Analysis of ATRX, IDH1 and p53 in Glioblastoma and Their Correlations with Patient Survival. J Korean Med Sci.

[15] Purkait S, Miller CA, Kumar A, Sharma V, Pathak P, Jha P, Sharma MC, Suri V, Suri A, Sharma BS, Fulton RS, Kale SS, Dahiya S (2017). ATRX in Diffuse Gliomas With its Mosaic/Heterogeneous Expression in a Subset. Brain Pathol.

[16] Rodriguez FJ, Vizcaino MA, Blakeley J, Heaphy CM (2016). Frequent alternative lengthening of telomeres and ATRX loss in adult NF1-associated diffuse and high-grade astrocytomas. Acta Neuropathol.

[17] Liu XY, Gerges N, Korshunov A, Sabha N, Khuong-Quang DA, Fontebasso AM, Fleming A, Hadjadj D, Schwartzentruber J, Majewski J, Dong Z, Siegel P, Albrecht S (2012). Frequent ATRX mutations and loss of expression in adult diffuse astrocytic tumors carrying IDH1/IDH2 and TP53 mutations. Acta Neuropathol.

[18] Heaphy CM, de Wilde RF, Jiao Y, Klein AP, Edil BH, Shi C, Bettegowda C, Rodriguez FJ, Eberhart CG, Hebbar S, Offerhaus GJ, McLendon R, Rasheed BA (2011). Altered telomeres in tumors with ATRX and DAXX mutations. Science.

[19] Cheung NK, Zhang J, Lu C, Parker M, Bahrami A, Tickoo SK, Heguy A, Pappo AS, Federico S, Dalton J, Cheung IY, Ding L, Fulton R (2012). Association of age at diagnosis and genetic mutations in patients with neuroblastoma. JAMA.

[20] Liau JY, Lee JC, Tsai JH, Yang CY, Liu TL, Ke ZL, Hsu HH, Jeng YM (2015). Comprehensive screening of alternative lengthening of telomeres phenotype and loss of ATRX expression in sarcomas. Mod Pathol.

[21] Koelsche C, Renner M, Johann P, Leiss I, Sahm F, Schimmack S, Wardelmann E, Renker EK, Schirmacher P, Korshunov A, von Deimling A, Mechtersheimer G (2016). Differential nuclear ATRX expression in sarcomas. Histopathology.

[22] Cancer Genome Atlas Research Network (2017). Comprehensive and Integrated Genomic Characterization of Adult Soft Tissue Sarcomas. Cell.

[23] Hagel C, Zils U, Peiper M, Kluwe L, Gotthard S, Friedrich RE, Zurakowski D, von Deimling A, Mautner VF (2007). Histopathology and clinical outcome of NF1-associated vs. sporadic malignant peripheral nerve sheath tumors. J Neurooncol.

[24] Reuss DE, Habel A, Hagenlocher C, Mucha J, Ackermann U, Tessmer C, Meyer J, Capper D, Moldenhauer G, Mautner V, Frappart PO, Schittenhelm J, Hartmann C (2014). Neurofibromin specific antibody differentiates malignant peripheral nerve sheath tumors (MPNST) from other spindle cell neoplasms. Acta Neuropathol.

[25] Prieto-Granada CN, Wiesner T, Messina JL, Jungbluth AA, Chi P, Antonescu CR (2016). Loss of H3K27me3 Expression Is a Highly Sensitive Marker for Sporadic and Radiation-induced MPNST. Am J Surg Pathol.

[26] Liu R, Kain M, Wang L (2012). Inactivation of X-linked tumor suppressor genes in human cancer. Future Oncol.

[27] Lee W, Teckie S, Wiesner T, Ran L, Prieto Granada CN, Lin M, Zhu S, Cao Z, Liang Y, Sboner A, Tap WD, Fletcher JA, Huberman KH (2014). PRC2 is recurrently inactivated through EED or SUZ12 loss in malignant peripheral nerve sheath tumors. Nat Genet.

[28] Zhang M, Wang Y, Jones S, Sausen M, McMahon K, Sharma R, Wang Q, Belzberg AJ, Chaichana K, Gallia GL, Gokaslan ZL, Riggins GJ, Wolinksy JP (2014). Somatic mutations of SUZ12 in malignant peripheral nerve sheath tumors. Nat Genet.

[29] Clynes D, Jelinska C, Xella B, Ayyub H, Scott C, Mitson M, Taylor S, Higgs DR, Gibbons RJ (2015). Suppression of the alternative lengthening of telomere pathway by the chromatin remodelling factor ATRX. Nat Commun.

[30] Napier CE, Huschtscha LI, Harvey A, Bower K, Noble JR, Hendrickson EA, Reddel RR (2015). ATRX represses alternative lengthening of telomeres. Oncotarget.

[31] Lee JC, Jeng YM, Liau JY, Tsai JH, Hsu HH, Yang CY (2015). Alternative lengthening of telomeres and loss of ATRX are frequent events in pleomorphic and dedifferentiated liposarcomas. Mod Pathol.

[32] Hirbe AC, Pekmezci M, Dahiya S, Apicelli AJ, Van Tine BA, Perry A, Gutmann DH (2014). BRAFV600E mutation in sporadic and neurofibromatosis type 1-related malignant peripheral nerve sheath tumors. Neuro Oncol.

