# Correction: Aberrant ATRX protein expression is associated with poor overall survival in NF1-MPNST

**DOI:** 10.18632/oncotarget.28137

**Published:** 2021-11-23

**Authors:** Hsiang-Chih Lu, Vanessa Eulo, Anthony J. Apicelli, Melike Pekmezci, Yu Tao, Jingqin Luo, Angela C. Hirbe, Sonika Dahiya

**Affiliations:** ^1^Division of Neuropathology, Department of Pathology and Immunology, Washington University School of Medicine, St. Louis, MO, USA; ^2^Department of Medicine, Washington University School of Medicine, St. Louis, MO, USA; ^3^Department of Radiation Oncology, Washington University School of Medicine, St. Louis, MO, USA; ^4^Siteman Cancer Center, Washington University School of Medicine, Saint Louis, MO, USA; ^5^Department of Pathology, University of California San Francisco School of Medicine, San Francisco, CA, USA; ^6^Siteman Cancer Center Biostatistics Shared Resource, Division of Public Health Sciences, Department of Surgery, Washington University School of Medicine, St. Louis, MO, USA; ^7^Division of Medical Oncology, Washington University School of Medicine, St. Louis, MO, USA; ^*^Co-first authors

**This article has been corrected:** The Supplementary Table 2 has been updated to show the appropriate WUSTL numbers in the first column. The corrected Table 2, produced using the original data, is shown below. The authors declare that these corrections do not change the results or conclusions of this paper.

Original article: Oncotarget. 2018; 9:23018–23028. 23018-23028. https://doi.org/10.18632/oncotarget.25195

**Supplementary Table 2:** Mitotic index

Case	Mitoses	Tumor Type
WU-1	74/10 HPF	MPNST
WU-2	17/10 PF	MPNST
WU-3	19/10 PF	MPNST
WU-4	28/10 HPF	MPNST
WU-5	78/10 HPF	MPNST
WU-6	4/10 HPF	MPNST
WU-7	6/10 HPF	MPNST
WU-8	5/10 HPF	MPNST
WU-9	81/10 HPF	MPNST
WU-10	11/10 HPF	MPNST
WU-11	^*^	MPNST
WU-12	25/10 HPF	MPNST
WU-13	3/10 HPF	MPNST
WU-14	8/10 HPF	MPNST
WU-15	25/10 HPF	MPNST
WU-16	32/10 HPF	MPNST
WU-17	27/10 HPF	MPNST
WU-18	^**^Metastatic lesion	MPNST
WU-19	9/10 HPF	MPNST
WU-20	27/10 HPF	MPNST
WU-21	6/10 HPF	MPNST
WU-22	8/10 HPF	MPNST
WU-23	6/10 HPF	MPNST
WU-24	4/10 HPF	MPNST
WU-25	3/10 HPF	MPNST
WU-26	23/10 HPF	MPNST
WU-27	15/10 HPF	MPNST
WU-28	4/10 HPF	MPNST
WU-29	20/10 HPF	MPNST
WU-30	21/10 HPF	MPNST
WU-31	69/10 HPF	MPNST
WU-32	9/10HPF	MPNST
WU-33	7/10 HPF	MPNST
WU-34	55/10 HPF	MPNST
WU-35	^**^Metastatic lesion	MPNST
WU-36	23/10 HPF	MPNST
WU-37	20/10 HPF	MPNST
WU-38	8/10 HPF	MPNST
WU-39	14/10 HPF	MPNST
WU-40	62/10 HPF	MPNST
WU-41	>1/50 HPF	Atypical Neurofibroma
WU-42	>1/50 HPF	Atypical Neurofibroma
WU-43	>1/50 HPF	Atypical Neurofibroma
WU-44	>1/50 HPF	Atypical Neurofibroma
WU-45	>1/50 HPF	Atypical Neurofibroma
WU-46	>1/50 HPF	Atypical Neurofibroma
WU-47	0 (small biopsy)	Atypical Neurofibroma
WU-48	0 (small biopsy)	Atypical Neurofibroma
WU-49	0 (small biopsy)	Atypical Neurofibroma

^*^Brisk mitotic activity per pathology report. All slides not available for current review. ^**^Mitotic count was not performed on metastatic lesions.

