# Supplementary material for: Aberrant ATRX protein expression is associated with poor overall survival in NF1-MPNST
Source: Oncotarget. 2018 May 1;9(33):23018–28. doi: 10.18632/oncotarget.25195 (PMC5955415; doi:10.18632/oncotarget.25195)
Supplement: Supplementary file 1 [file oncotarget-09-23018-s001.pdf]

Aberrant ATRX protein expression is associated with poor overall survival in NF1-MPNST

SUPPLEMENTARY MATERIALS

Supplementary Table 1: Correlation between ATRX IHC and mutation status in a subset of MPNSTs

Patient number	ATRX stain	*ATRX* mutation	*TP53* mutation	*CDKN2A* mutation	*NF1* mutation	*EGFR* mutation	*PDGFRA* mutation	*EED* or *SUZ 12* mutation
6	Loss	Yes	Yes	No	Yes	No	No	No Data
8	Loss	Yes	No	No	Yes	Yes	Yes	No Data
11	Retained	No	No	No	Yes	No	No	No Data
12	Retained	No	No	No	Yes	No	No	No Data
14	Retained	No	No	No	Yes	No	No	No Data
15	Retained	No	Yes	No	Yes	No	No	No Data

Supplementary Table 2: Mitotic index. See Supplementary_Table_2
