# Supplementary material for: Aberrant ATRX protein expression is associated with poor overall survival in NF1-MPNST
Source: Oncotarget. 2018 May 1;9(33):23018–28. doi: 10.18632/oncotarget.25195 (PMC5955415; doi:10.18632/oncotarget.25195)
Supplement: Supplementary file 2 [file oncotarget-09-23018-s002.docx]

**Supplementary Table 2: Mitotic Index**

| **Case** | **Mitoses** | **Tumor Type** |
| --- | --- | --- |
| \| S01-24629 \|  \| \| --- \| --- \| | 74/10 HPF | MPNST |
| S03-1024 | 17/10 PF | MPNST |
| S03-37775 | 19/10 PF | MPNST |
| S04-28380 | 28/10 HPF | MPNST |
| S04-32241 | 78/10 HPF | MPNST |
| S06-10157 | 4/10 HPF | MPNST |
| S06-20947 | 6/10 HPF | MPNST |
| S06-46769 | 5/10 HPF | MPNST |
| S06-12345 | 81/10 HPF | MPNST |
| S06-12349 | 11/10 HPF | MPNST |
| S06-26020 | * | MPNST |
| S07-11266 | 25/10 HPF | MPNST |
| S07-08343 | 3/10 HPF | MPNST |
| S07-11937 | 8/10 HPF | MPNST |
| S07-29018 | 25/10 HPF | MPNST |
| S07-33966 | 32/10 HPF | MPNST |
| S07-9667 | 27/10 HPF | MPNST |
| S08-11969 | **Metastatic lesion | MPNST |
| S08-49351 | 9/10 HPF | MPNST |
| S09-14361 | 27/10 HPF | MPNST |
| S09-19633 | 6/10 HPF | MPNST |
| S09-25723 | 8/10 HPF | MPNST |
| S09-28110 | 6/10 HPF | MPNST |
| S09-4600 | 4/10 HPF | MPNST |
| S10-2054 | 3/10 HPF | MPNST |
| S10-42953 | 23/10 HPF | MPNST |
| S12-43374 | 15/10 HPF | MPNST |
| S12-45485 | 4/10 HPF | MPNST |
| S12-50469 | 20/10 HPF | MPNST |
| S13-15752 | 21/10 HPF | MPNST |
| S13-28362 | 69/10 HPF | MPNST |
| S13-29497 | 9/10HPF | MPNST |
| S13-7627 | 7/10 HPF | MPNST |
| S14-11190 | 55/10 HPF | MPNST |
| S14-20839 | **Metastatic lesion | MPNST |
| S14-22657 | 23/10 HPF | MPNST |
| S15-39894 | 20/10 HPF | MPNST |
| S16-12325 | 8/10 HPF | MPNST |
| S16-13806 | 14/10 HPF | MPNST |
| S16-15081 | 62/10 HPF | MPNST |
| S06-26393 | >1/50 HPF | Atypical Neurofibroma |
| S06-42721 | >1/50 HPF | Atypical Neurofibroma |
| S07-43610 | >1/50 HPF | Atypical Neurofibroma |
| S12-29326 | >1/50 HPF | Atypical Neurofibroma |
| S12-41426 | >1/50 HPF | Atypical Neurofibroma |
| S14-11851 | >1/50 HPF | Atypical Neurofibroma |
| S16-33726 | 0 (small biopsy) | Atypical Neurofibroma |
| S17-26369 | 0 (small biopsy) | Atypical Neurofibroma |
| S14-23830 | 0 (small biopsy) | Atypical Neurofibroma |

*Brisk mitotic activity per pathology report. All slides not available for current review. **Mitotic count was not performed on metastatic lesions.
